# Diversity of the gut, vaginal and oral microbiome among pregnant women in South Africa with and without pre-eclampsia

**DOI:** 10.3389/fgwh.2022.810673

**Published:** 2022-09-16

**Authors:** Janri Geldenhuys, Mathys J. Redelinghuys, Hendrik A. Lombaard, Marthie M. Ehlers, Don Cowan, Marleen M. Kock

**Affiliations:** ^1^Department of Medical Microbiology, Faculty of Health Sciences, University of Pretoria, Pretoria, South Africa; ^2^Centre for Microbial Ecology and Genomics, Department of Biochemistry, Genetics and Microbiology, University of Pretoria, Pretoria, South Africa; ^3^Obstetrics and Gynecology, Rahima Moosa Mother and Child Hospital, Wits Obstetrics and Gynecology Clinical Research Division, School of Clinical Medicine, Faculty of Health Sciences, University of Witwatersrand, Johannesburg, South Africa; ^4^Department of Medical Microbiology, Tshwane Academic Division, National Health Laboratory Service, Pretoria, South Africa

**Keywords:** gut microbiome, vaginal microbiome, oral microbiome, pre-eclampsia, pregnancy, 16S rRNA sequence, diversity

## Abstract

**Background:**

Changes in microbial communities are a known characteristic of various inflammatory diseases and have been linked to adverse pregnancy outcomes, such as preterm birth. However, there is a paucity of information regarding the taxonomic composition and/or diversity of microbial communities in pre-eclampsia. The aim of this study was to determine the diversity of the gut, vaginal and oral microbiome in a cohort of South African pregnant women with and without pre-eclampsia. The diversity of the gut, vaginal and oral microbiome was determined by targeted next generation sequencing (NGS) of the V3 and V4 region of the 16S rRNA gene on the Illumina MiSeq platform.

**Results:**

In this study population, pre-eclampsia was associated with a significantly higher alpha diversity (*P* = 0.0472; indicated by the Shannon index) in the vaginal microbiome accompanied with a significant reduction in *Lactobacillus* spp. (*P* = 0.0275), compared to normotensive pregnant women. *Lactobacillus iners* was identified as the predominant species of the vaginal microbiome in both cohorts. High inter-individual variation in alpha diversity was observed in the gut and oral microbiome in both cohorts. Although differences in the relative abundance of bacteria at all phylogenetic levels were observed, overall microbial composition of the gut, oral and vaginal microbiome was not significantly different in the pre-eclampsia cohort compared to the normotensive cohort.

**Conclusion:**

Collectively, a reduction of *Lactobacillus* spp., and predominance of *L. iners* in pregnant women with pre-eclampsia could suggest an unstable vaginal microbiome that might predispose pregnant women to develop pre-eclampsia. The lack of significant structural changes in the gut, oral and vaginal microbiome does not suggest that the characterized communities play a role in pre-eclampsia, but could indicate a characteristic unique to the study population. The current study provided novel information on the diversity of the gut, oral and vaginal microbiome among pregnant women in South Africa with and without pre-eclampsia. The current study provides a baseline for further investigations on the potential role of microbial communities in pre-eclampsia.

## Background

Pre-eclampsia is a multisystem and multifactorial disorder unique to pregnancy, diagnosed by new-onset hypertension during pregnancy and damage to one or more organ systems featuring proteinuria, hemolysis, elevated liver enzymes and low platelet count, neurological or visual symptoms, abnormal Doppler ultrasound and fetal growth restriction (1–3). This disorder is further characterized by two subtypes known as the maternal subtype, with metabolic and immunological involvement, and the placental subtype characterized by an ischemic placenta followed by maternal systemic inflammation (4, 5). Pre-eclampsia has a complex etiology for which diagnostic biomarkers and approved therapies are not yet available. The exact cause of this condition remains unknown; however, its association with exaggerated systemic inflammation and certain risk factors, such as diabetes and obesity, has suggested the microbiome may play a role in disease pathogenesis (6–8). The maternal microbiome is considered an important factor that is affected by and which influences the physiological processes in pregnancy including immune, metabolic and hormonal changes (9). Microbial communities in the placenta, vagina, the distal gut and the oral cavity have previously been implicated in maternal health and potentially also in the pathogenesis of pre-eclampsia (10–12). Since Amarasekara et al. (13) described the placental microbiome in a group of pregnant women with pre-eclampsia, conflicting information on the presence and potential role of a placental microbiome in pre-eclampsia have been reported (14–18). However, the presence of commensal microorganisms originating from the oral cavity, gut and vagina in the placenta of pre-eclampsia and hypertensive pregnancies has refocused the role of the placental microbiome by means of bacterial translocation (12, 13, 19).

Dysbiosis of the gut microbiome may contribute to the pathogenesis of hypertension and affect maternal adaptation in pregnancy including placental function (20, 21). The contributing role of a disrupted gut microbiome in metabolic disease, blood pressure (BP) regulation, chronic inflammatory diseases and complicated pregnancies point to a possible role in the development of pre-eclampsia (19, 22, 23). Several studies investigated the composition and stability of the vaginal microbiome in reproductive-age women, uncomplicated pregnancy and in pregnant women with spontaneous preterm delivery (24–26). The vaginal microbiome in uncomplicated pregnancy is characterized by lower richness and diversity with an increase in abundance of the *Lactobacillus* species, to obtain stability and resilience during pregnancy (27). However, there is uncertainty on the composition and contributing role of the vaginal microbiome in preterm birth. Although a reduction in *Lactobacillus* species has been suggested as a risk factor for preterm delivery, different findings have also been reported (25, 26, 28–30).

The oral microbiome is known as one of the most diverse microbiomes in the human body with a strong impact on systemic health (31–33). Periodontal disease can cause systemic illness including atherosclerotic cardiovascular disease, rheumatoid arthritis, diabetes and adverse pregnancy outcomes (34–39). Bacterial species associated with oral infections have the ability to translocate and colonize extra-oral sites such as the placenta, and this phenomenon has led to the establishment of a link between oral infections and adverse systemic conditions (40–42). In addition, maternal periodontal disease has previously been found to be associated with an increased risk of preterm birth and pre-eclampsia (43–45).

Currently available next generation sequencing (NGS) techniques, including 16S targeted rRNA sequencing and metagenomics enable the characterization of microbial communities associated with pregnancy and pregnancy complications, such as pre-eclampsia. The characterization of microbial communities in pre-eclampsia may reveal whether structural changes in microbial communities are associated with pre-eclampsia and may also provide indicative biomarkers for the development of future preventative and therapeutic strategies. The aim of this study was to determine the diversity of the gut, vaginal and oral microbiome in a cohort of pre-eclampsia and normotensive pregnant women.

## Methods

### Sample collection

All participants in this study were recruited from a provincial hospital in Johannesburg, South Africa. Ten primiparous pregnant women, ≥18 years and with a gestational age ≥28 gestational weeks (3rd trimester), were included in either one of two cohorts (*N* = normal/normotensive and *P* = pre-eclampsia) following the rule of ten (46). Participants were selected according to the study criteria after a medical examination was done by a healthcare practitioner or using hospital records when available. Participants who had symptomatic vaginal infections including known viral infections, such as human papilloma virus (HPV), antibiotic usage in the 4 weeks prior to sample collection, previous miscarriages and a gestational age ≤ 28 weeks were excluded from the study. Informed consent was obtained prior to sample collection.

Three samples were collected from each enrolled participant to characterize the gut, vaginal and oral microbiome, respectively. A rectal swab as an alternative to a stool sample was collected by an obstetrician by inserting a flocked dry swab (FLOQSwabs 552C, Copan, CA) into the anal canal (±3 cm) beyond the anal verge (47). A midvaginal swab was collected by the insertion of a dry flocked swab into the vaginal canal (±2 cm) to absorb vaginal fluid. Saliva was obtained by the use of a sterile flocked swab in a stable position under the tongue for 2 min. Fresh collected samples were labeled, kept and transported on ice to the laboratory of the Department of Medical Microbiology, University of Pretoria, Pretoria and stored at −20°C until processing. The sample labeling used in this study refers to the site of collection, i.e., R = rectal/gut, V = vaginal, O = oral sample in both cohorts.

### Bacterial genomic DNA isolation

The extraction methods used in this study were adapted with the use of different enzymes for bacterial lysis and sufficient isolation of all bacterial organisms present in the gut, oral cavity and the vagina (24, 48, 49). The stored dry swabs (−20°C; F700-SAEV-TSC; Thermo Scientific, Waltham, MA) were thawed on ice for 30 min prior to genomic DNA isolation. A volume of 2 mL sterile 1X phosphate buffered saline (PBS) (Life Technologies Corporation, Carlsbad, CA, USA) was added to thawed samples. Rectal and vaginal swabs were incubated at room temperature (25°C ± 5°C) for 2–3 h and mixed in a VX-100 vortex mixer (Labnet International, Edison, NJ) at maximum speed for 5 min to efficiently suspend all cellular material (24). Oral swabs were incubated overnight at 4°C and ere mixed vigorously at maximum speed for 5 min to release all cellular material into the PBS solution (24). Total bacterial genomic DNA (gDNA) was isolated using the Bioline ISOLATE II Genomic DNA kit (Bioline) following the manufacturer's instructions with minor modifications (see Supplementary material). The concentration of the isolated gDNA was determined by using the NanoDrop^R^ ND-1000 Spectrophotometer (Thermo Scientific) and the purity was determined by the ratio of absorbance at 260 and 280 nm (A_260/280_). The isolated DNA was normalized to a final concentration of 10 ng/μL in a total volume of 25 μL. The normalized DNA was used for 16S rRNA amplification and sequencing.

### Targeted amplification of the 16S rRNA variable domains (V3 and V4)

A genome sequence spanning the V3 and V4 regions of the 16S rRNA gene were amplified using the Illumina ultramer oligonucleotides (Integrated DNA Technologies, Coralville, IA) that include Illumina adapter overhang nucleotide sequences (50). Polymerase chain reaction (PCR) conditions and reagent volumes are available as Supplementary Information. A negative control from each gDNA isolation step was included in amplification steps to determine the possibility of carry-over contamination between samples and reagents during these steps. Amplification products were purified using the ISOLATE II PCR and Gel Kit (Bioline) according to the manufacturer's instructions with minor adjustments (see Supplementary material).

### Library preparation and 16S targeted NGS

The purified amplified products were submitted to the ARC Biotechnology Platform, Onderstepoort, Pretoria, South Africa for library preparation and NGS on the Illumina MiSeq platform. Sequencing libraries were created according to the Illumina 16S metagenomic library preparation protocol available online (see Supplementary material). The 16S rRNA gene was sequenced on a MiSeq sequencer creating 300 bp paired-end reads with the V3 600 cycles kit (Illumina, San Diego, CA). Raw paired-end FASTQ files of each sample were received for subsequent analysis.

### Sequence and statistical analysis

The targeted metagenomic sequence data were analyzed using the Quantitative Insights Into Microbial Ecology (QIIME) pipeline, version 9.1 (51). Matching paired-end sequences were merged using the *fastq-join* command in the *ea-utils* software package (52). Chimeric sequences were identified from merged sequences and removed using the USEARCH database version 6.1 (53). The open reference method was used to assign the sequences to 97% identity (ID) operational taxonomic units (OTUs). Taxonomy was assigned when the representative sequence of each OTU was aligned against the GreenGenes database 12_10, using the Basic Local Alignment Search Tool (BLAST) program incorporated into the QIIME software (54).

The resulting unrarefied OTU table and phylogenetic tree generated in QIIME were used for diversity and statistical analysis done using Rstudio version 1.0.136 (55). Statistical analysis and the generation of graphs using *ggplots* (integrated in the package *phyloseq* version 1.19.1 and the package *tidyverse* version 1.1.1) were used as implemented in Rstudio. These packages were also used to generate stacked bar plots for visualization of the taxonomic composition in each sample. To select only sequences representing bacteria, sequences representing mitochondria, *Archaea* and chloroplast were removed from the OTU table and phylogenetic tree in Rstudio. Samples that contained fewer than the rarefaction depth of 1,021 OTUs were removed for alpha and beta diversity analysis. The resulting dataset included a total of fourteen samples in the gut microbiome, ten samples in the vaginal microbiome and eleven samples in the oral microbiome. The data of each microbiome were extracted from the rarefied OTU table to infer statistical analysis of each microbiome individually. Due to the small sample size, it was assumed that the data do not follow a normal distribution pattern. Therefore, the non-parametric Kruskal-Wallis rank chi-squared test was used to evaluate differences (*P*-value < 0.05) in alpha diversity and the relative percent abundances of taxa between cohorts with a 95% confidence interval (56). Alpha diversity was measured by the Chao1 index, an indirect measure of richness and Shannon's diversity index, an indirect measure of evenness (56).

In beta diversity analysis, principal coordinate analysis (PCoA) plots were generated with weighted and unweighted unifrac distance matrices to vizualize the differences between cohorts (57). The statistical significance in the differences in microbial communities between the cohorts was calculated using a permanova test, *Adonis* (Analysis of Dissimilarity), as implemented in the R package *vegan* version 2.4–3.0 (58). This method included 999 permutations and the weighted and unweighted unifrac distance matrices.

## Results

Samples were obtained from 21 participants who provided informed consent and who met the inclusion criteria. Eleven participants were recruited in the control group and ten participants were recruited to the study group. All participants were in an age group between 18 and 35 years with a gestational age between 32 and 40 weeks. DNA extracted from each sample was quantified, followed by amplification and then used for subsequent analysis. In the sequence analysis, the selection of OTUs in QIIME resulted in a dataset of 545,677 sequences and 1,751 identified taxa between the 63 samples. An average of 9,094 OTUs were formed with a minimum of 73 and a maximum of 64,247 OTUs between 63 samples. Rarefaction prior to statistical analysis allowed normalization of sequencing data and identification of samples with insufficient sequencing data; insufficient sequencing data could affect subsequent analysis due to inaccurate representation of microbial communities. After rarefaction, the OTU table including the gut, vaginal and oral microbiome consisted of 884 taxa and 35 samples in total (reduced from an initial 63 samples). The rarefied cohort consisted of a total of 14 samples (normotensive, *N* = 8; pre-eclampsia, *P* = 6) for analysis of the gut microbiome, 10 samples (*N* = 5; *P* = 5) of the vaginal microbiome and 11 samples (*N* = 5; *P* = 6) of the oral microbiome. The 35 samples on which all analyses were carried out come from a pool of 14 participants in the final cohort (i.e., three and four participants were excluded from the normotensive and pre-eclampsia cohorts, respectively, as none of their samples were included after rarefaction). All participants were of black South African ethnicity except one of the participants who was of white South African ethnicity.

### Alpha and beta diversity analysis

**Figure 1:**
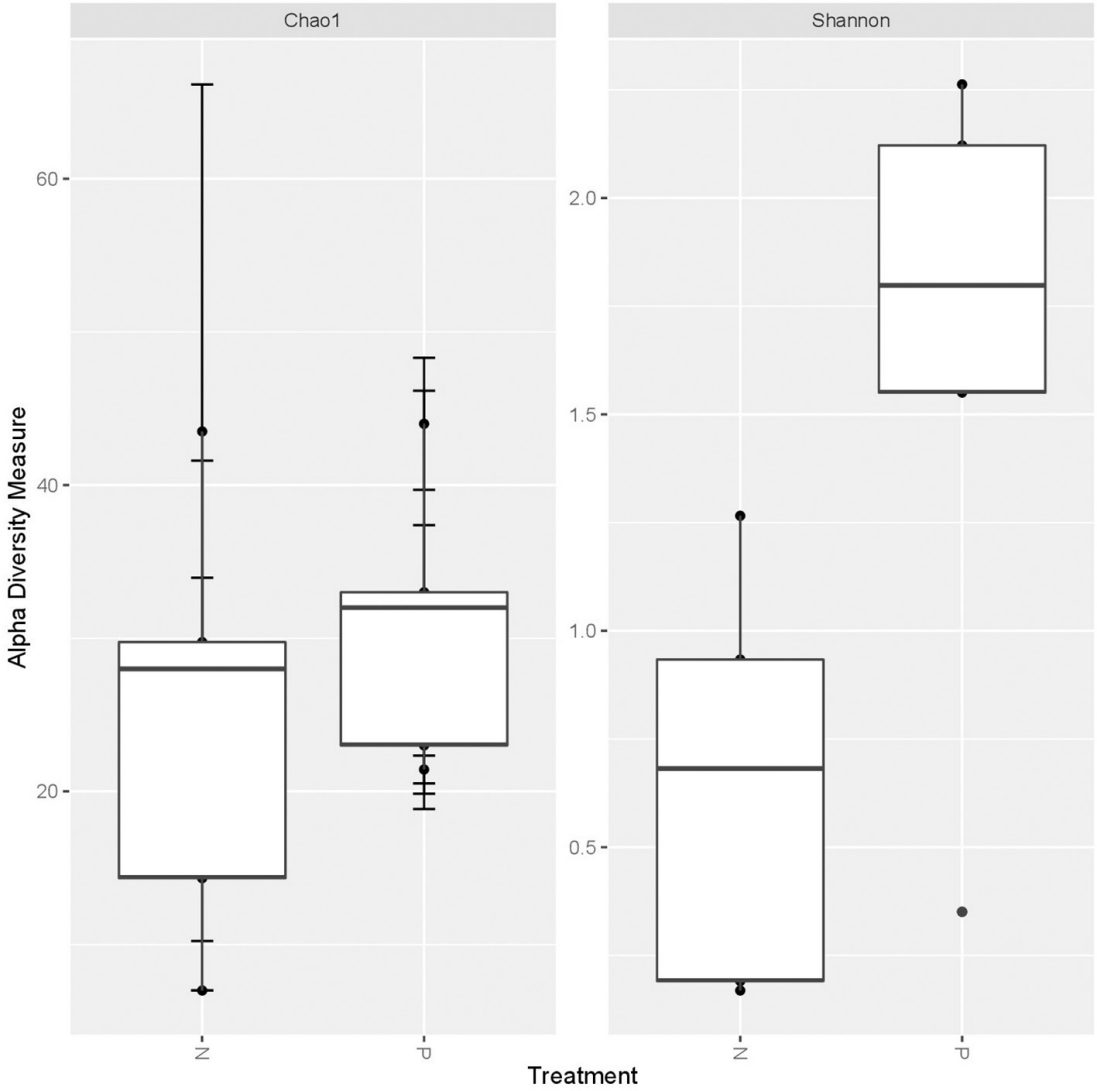
Box-plot illustrating alpha diversity indices (Chao1 and Shannon diversity index) of the vaginal microbiome comparing the normotensive (*n* = 5) and pre-eclampsia (*p* = 5) cohort.

A higher average alpha diversity was found in the gut, oral and vaginal microbiome in pregnant women with pre-eclampsia in comparison to normotensive pregnant women (Table 1). The differences in alpha diversity observed between cohorts in the gut microbiome was not significant for both the Chao1 index (*P* = 0.1213) and Shannon index (*P* = 0.09329). Differences were also not significant for both the Chao1 (*P* = 1) and the Shannon index (*P* = 0.715) in the oral microbiome. In the vaginal microbiome, a significant difference in diversity was observed between the two groups (*P* = 0.0472) for the Shannon index, but not the Chao1 index (*P* = 0.3472) (Figure 1).

**Table 1:** The average alpha diversity measures of the gut, vaginal and oral microbiome as assessed by the Chao1 richness Index and the Shannon Index.

**Microbiome**	**Cohort**	**Chao1 richness**	**Shannon diversity**
Gut	N	128.67	3.38
	P	208.95	3.89
Vaginal	N	24.5	0.64
	P	30.68	1.61
Oral	N	118.72	2.18
	P	121.91	2.45

**Figure 2:**
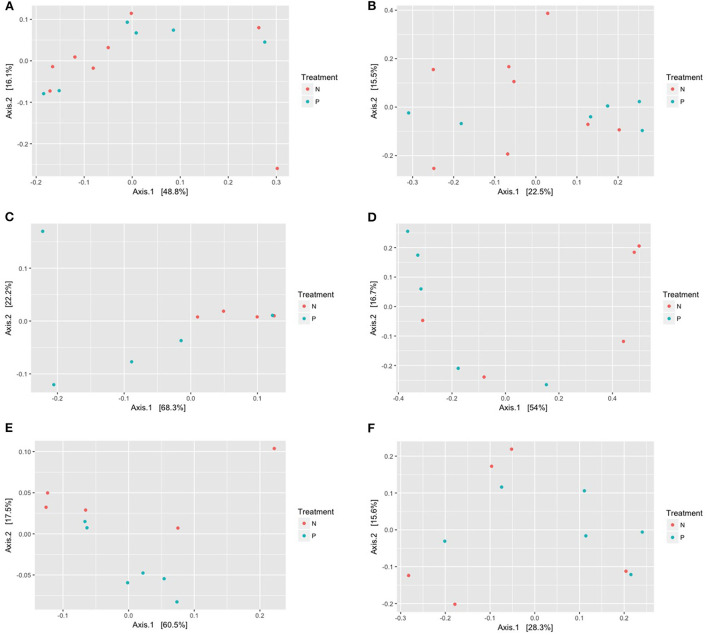
**(A–F)** Principal coordinate analysis (PCoA) of the gut, vaginal, and oral microbiome displaying the dissimilarities among the gut microbiome in the normotensive group compared to the pre-eclampsia group using the weighted **(A,C,E)** and the unweighted **(B,D,F)** UniFrac distance metrices. Each point indicates the microbiome of each sample in the *N* = normotensive cohort and *P* = pre-eclampsia cohort.

Principal coordinate analysis based on the UniFrac distances using phylogenetic information revealed no distinct separation of microbial communities of the gut, vaginal and oral microbiome amongst both cohorts (Figures 2A–F). The dissimilarity test, *Adonis*, indicated that the gut microbiome in the pre-eclampsia group is not significantly different compared to that of the normotensive cohort using both the UniFrac weighted (*R*^2^ = 0.02266; *P* = 0.987) and unweighted (*R*^2^ = 0.07072; *P* = 0.568) distance matrices. Similarly, the oral microbiome of the pre-eclampsia group was also not significantly different to that of the normotensive cohort using the UniFrac weighted (*R*^2^ = 0.10288, *P* =0.354) and unweighted (*R*^2^ = 0.12881, *P* = 0.185) distance matrices. In the vaginal microbiome, a clear separation of the pre-eclampsia and normotensive cohort was visible on the PCoA plot with the UniFrac weighted distance matrix; however, the finding was not statistically significant with both the weighted (*R*^2^ = 0.30017, *P* = 0.05) and unweighted distance matrix (*R*^2^ = 0.2344, *P* = 0.068) (Figure 2C).

### Comparison of the gut microbiome between cohorts

The gut microbiome of both groups was analyzed at different taxonomic levels including at phylum, genus and species level. Although the *Firmicutes* was the most abundant phylum, the relative abundance did not differ significantly between cohorts (*Firmicutes*; *P* = 0.6985, *Bacteroidetes*; *P* = 0.8973, *Fusobacteria*; *P* = 0.08117, *Proteobacteria*; *P* = 0.7026, *Actinobacteria*; *P* = 0.9485). Significant differences of relative abundance were not observed on genus level (*P* values > 0.05) between cohorts; however, changes in the relative abundance of several genera were observed. The pre-eclampsia group had a higher relative abundance of *Bacteroides* (6.41% as opposed to 4.73%)*, Faecalibacterium* (12.35% as opposed to 10.61%) and *Blautia* (7.61% as opposed to 3.67%). In contrast, the normotensive group had a higher relative abundance of *Anaerococcus* (7.56% as opposed to 3.59%)*, Clostridioides* (5.64% as opposed to 3.21%), *Finegoldia* (4.78% as opposed to 2.9%) and *Prevotella* (15.17% as opposed to 12.30%).

On species level, a higher relative abundance of *Peptostreptococcus anaerobius* (average relative abundance of 23.36%) was observed in the pre-eclampsia group compared to the normotensive group (7.8%), although the difference was not significant (*P* = 0.1824). *Faecalibacterium prausnitzii* (average relative abundance of 42.69 and 35.79%, respectively) and *Lactobacillus iners* (average relative abundance of 36.49 and 21.67%, respectively) were more abundant in the pre-eclampsia group compared to the normotensive group.

### Comparison of the vaginal microbiome between cohorts

**Figure 3:**
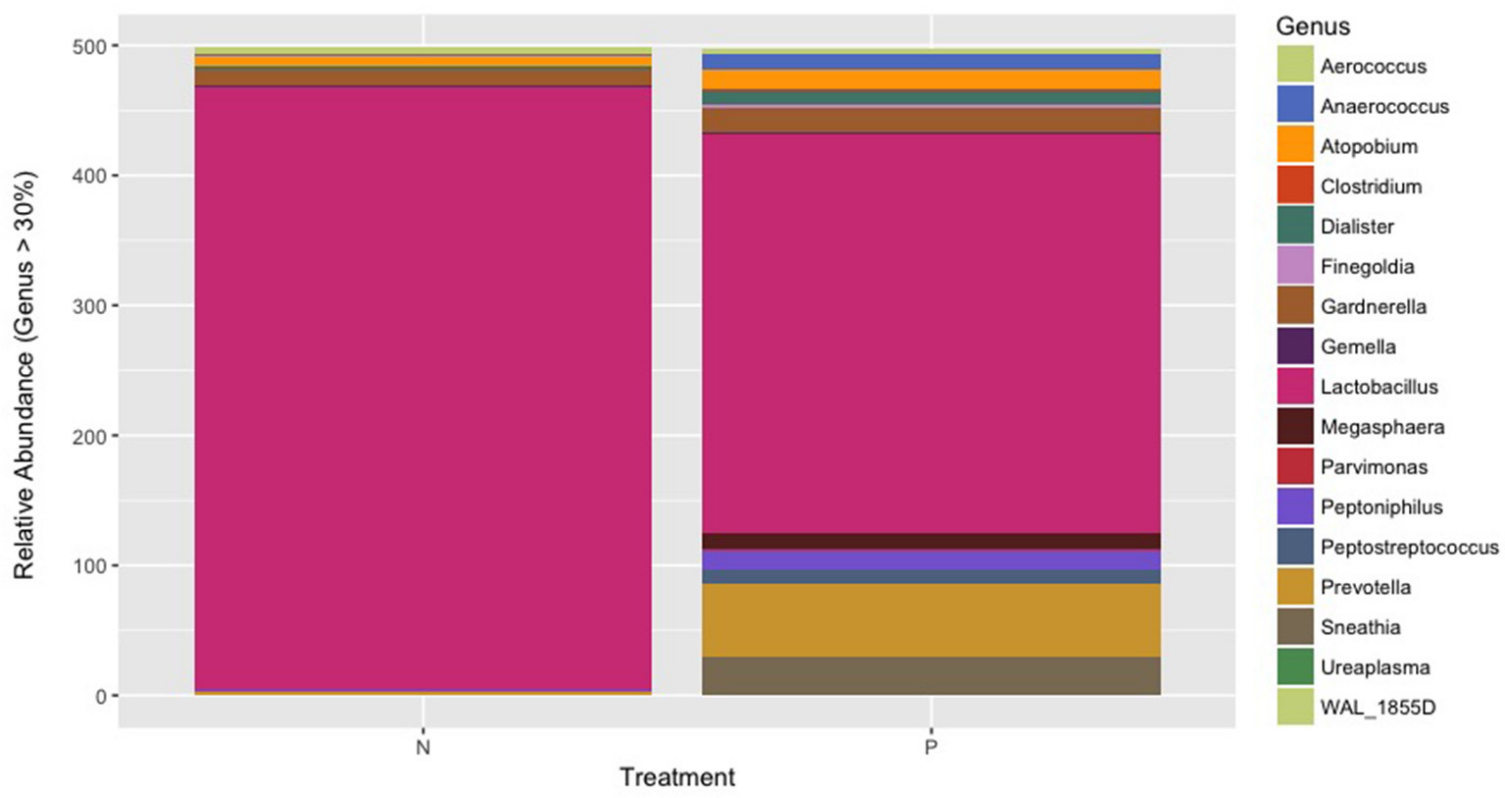
The overall microbial composition of the vaginal microbiome present at genus level showing the genera present in more than 30% of each sample in both the normotensive cohort and the pre-eclampsia cohort.

The *Firmicutes* was the predominant phylum in the vaginal microbiome with an average relative abundance of 95.2 and 74.28% in normotensive and pre-eclampsia cohort, respectively. Figure 3 indicates the overall microbial composition at genus level. Only genera with a relative abundance of more than 30% in each sample is indicated on the plot. The genus *Lactobacillus* was significantly lower (*P* = 0.0275) in the pre-eclampsia cohort (61.5%) compared to the normotensive cohort (92.6%). Higher relative abundances of the genera *Prevotella, Peptoniphilus* and *Anaerococcus* and an increase in *Dialister* were also observed in the pre-eclampsia cohort. The genera *Sneathia, Parvimonas, Clostridium, Megasphaera* and *Peptostreptococcus* were uniquely detected in only the pre-eclampsia cohort (Figure 3).

Present in low levels of abundance, *Atopobium* spp. were present in equal relative abundance in both groups (3.93 and 3.89%), whereas a slight increase in *Aerococcus* spp. (average relative abundance of 2.94% compared to 1.92%) and *Gardnerella* spp. (5.84% average relative abundance compared to 4.83%) was detected in the normotensive group. *Lactobacillus iners* was identified as the predominant species in the vaginal microbiome of both the normotensive (97.71%) and pre-eclampsia cohort (86.14%). A higher relative abundance of *Atopobium vaginae* (average relative abundance of 6.56% compared to 4.51%) was observed in the pre-eclampsia cohort. The presence of *Prevotella melaninogenica* and *Peptostreptococcus anaerobius* were also uniquely detected in the pre-eclampsia cohort.

### Comparison of the oral microbiome between cohorts

The PCoA of the oral microbiome illustrated in Figures 2E,F indicated no clear separation of the oral microbial communities between normotensive pregnant women and women with pre-eclampsia. However, this plot also displayed the similarity and high variation within cohorts, which was confirmed by the relative abundance of oral bacteria on genus and species level. The *Firmicutes* was the predominant phylum in the oral microbiome among the normotensive (70.98%) and pre-eclampsia cohort (67.76%) respectively. On genus level, *Streptococcus* was the predominant bacterial genus in both cohorts followed by order of decreasing abundance of *Haemophilus, Neisseria, Prevotella* and *Veillonella*. Higher relative abundance values of *Streptococcus, Granulicatella* and *Veilonella* were observed in the normotensive group. *Neisseria* (4.46 and 4.27%) and *Porphyromonas* (2.49 and 2.68%) were detected in similar relative abundance in both cohorts. *Prevotella* (5.16 and 4.13%) and *Haemophilus* (13.29% compared to 8.4%) were more abundant in the pre-eclampsia cohort. Genera such as *Rothia, Sneathia, Treponema, Parvimonas, Prevotella, Pseudomonas* and *Gemella* were detected in low abundance.

The pre-eclampsia cohort had higher average relative abundance of the species *Prevotella melaninogenica* (23.57%) and *Neisseria subflava* (6.96%) compared to the normotensive cohort (17.84%, 3.93% respectively). *Neisseria cinerea* and *N. Oralis* were detected in both cohorts. The relative abundance of the species *Rothia dentocariosa* was significantly higher (*P* = 0.02535) in the normotensive cohort compared to the pre-eclampsia cohort.

## Discussion

To our knowledge, this is the first study characterizing and determining the diversity of the gut, vaginal and oral microbiomes among pregnant women in South Africa with pre-eclampsia. Pre-eclampsia is a complication of hypertension in pregnancy and is one of the main contributions to the high maternal/fetal morbidity and mortality rates globally as well as in South Africa (59, 60). Research to uncover the complicated pathology involved in pre-eclampsia is ongoing in order to develop preventative and therapeutic strategies. In the current study, pregnant women with pre-eclampsia displayed a greater phylogenetic diversity in terms of richness (the number of taxa present) and evenness (abundance of many microbial constituents) compared to normotensive pregnant women. High inter-individual variation was evident in the characterization of the gut and oral microbiome and with a lesser extent in the vaginal microbiome and significant structural changes of microbial communities were not found. The high inter-individual variation in this study is supported by the observations of the Human Microbiome Project Consortium, which reported that individuals differ with the carriage of specific microbes and that inter-individual variation in the microbiome is specific, functionally relevant and personalized (61). Regardless of inter-individual variation and small sample size, a significant increase in alpha diversity accompanied with a reduction in *Lactobacillus* species was found in the vaginal microbiome of this cohort of pregnant women with pre-eclampsia, in comparison to normotensive pregnant women.

A decrease in gut microbial diversity is a known suggestive indicator of microbial imbalance associated with human disease (62). In contrast to the findings of the current study (19), found a decrease in alpha diversity in pregnant women with pre-eclampsia. Another study found no differences in diversity between pregnant women with and without pre-eclampsia (63). Alpha diversity analysis of the current study is therefore not suggestive of an imbalanced or disrupted gut microbiome. As indicated by Frost et al. (62), an increase in alpha diversity could rather contribute to greater stability and resilience of the gut microbiome. Taxonomic characterization of the gut microbiome revealed a mixed species microbiome with the exception of a few samples having a single dominant bacterial species, including *Bifidobacterium longum, Moryella indoligenes* and *Lactobacillus iners*. Diversity and composition of gut microbiome is versatile and is influenced by various external and internal factors such as diet and host genetics. Species dominance could be specific to an individual at a specific time with presence relating to a species specific function (64). Several species with anti-inflammatory properties such as *Faecalibacterium prausnitzii* was detected in the pre-eclampsia cohort, indicated a balanced gut microbiome with no significant structural changes to play a possible role in the development of pre-eclampsia (65–69).

Similarly to the gut microbiome, a diverse oral microbiome was characterized in women with and without pre-eclampsia. Diversity of the oral microbiome has been found to be stable during pregnancy and has also not been found to change in oral disease (70). Changes in alpha diversity is therefore not suspected to play a role in pre-eclampsia. Microbial characterization indicated dominance of *Streptococcus* spp., that has been reported in healthy oral microbiomes (71, 72). Other species, such as *Rothia, Haemophilus and Neisseria* spp., have been observed in healthy individual and therefore it is unknown whether these species could have contributed to pre-eclampsia in the current study (73–75). The presence of these species indicates a diverse oral microbiome present in pregnant women with and without pre-eclampsia. In addition, no pathogenic species associated with periodontitis were detected in the current study to hypothesize any link with pre-eclampsia (76).

Characterization of the vaginal microbiome in both cohorts has provided more information on the structure of vaginal microbial communities of South African pregnant women. Racial variation has a strong influence on community structure; for example, *Aerococcus* spp., *Peptoniphilus* spp., *Dialister* spp., *Atopobium* spp., *Sneathia* spp. and *Gardnerella* spp., have been detected more among healthy African American and African women (77, 78). In addition, a less acidic vaginal environment described in black and Hispanic women could also influence microbial diversity (24). Results within the South African population have similarly reported dominance of *L. iners* in black South Africa women (79). *Lactobacillus iners* has also been reported to co-exist with other BV-related bacteria (24, 78, 80–83).

Taxonomic observations in the pre-eclampsia cohort showed similarities to a BV-associated vaginal microbiome, which is characterized by higher bacterial diversity, lower abundances of *Lactobacillus* spp. and higher abundances of *Atopobium* spp., *Dialister* spp., *Gardnerella* spp., *Prevotella* spp. and *Sneathia* spp. (84). Low abundances of *Lactobacillus* spp., may have significant health consequences such as increase vaginal inflammation and susceptibility to pathogenic infection (79). A decrease in relative abundance of *Lactobacillus* spp., together with dominance of *L. iners* could further contribute to an unstable vaginal microbiome in women with pre-eclampsia. Petricevic et al. (85) described a vaginal microbiome dominated by a single vaginal *Lactobacillus* species, specifically *L. iners* in the late first trimester of pregnancy that might have been associated with preterm birth. Another study by Kindlinger et al. (86) identified the predominance of *L. iners* as a risk factor for preterm birth, while the predominance of *L. crispatus* was highly predictive of a term birth. Other studies found *L. iners* to represent a transitional phase of the vaginal microbiome between healthy and dysbiotic states that may promote the recurrence of BV (80, 87–89).

Regardless of low sample numbers, the results of the current study align with previous studies that found an increase in bacterial diversity accompanied by a reduction in *Lactobacillus* spp., in complicated pregnancies (11, 86, 90–92). Diversity analysis by the weighted UniFrac distance matrix suggested that the relative abundance of bacterial taxa may play a more important role in comparative analysis than the absence or presence of taxa between the two groups. The co-existence of *L. iners* and anaerobic taxa in both cohorts could indicate an unstable and transitional vaginal microbiome in South African pregnant women (78, 93–95). However, these findings concurrently with a significant reduction of *Lactobacillus* spp., found in pregnant women with pre-eclampsia could discriminate a vaginal microbiome unique to a study population to a vaginal microbiome that could increase the risk of developing pre-eclampsia.

The strengths of this study were the identification of microbial taxa on all phylogenetic levels, especially on species level in low percentage abundances, which is needed to unlock the functional complexity of a microbiome in health and disease. Analysis at species level is critical as different species may have different functional capacities, which is needed to link changes in the human microbiome to health or disease. The sample collection strategy used in this study accounted for as much variability as possible by including a homogenous group of pregnant women in their third trimester of pregnancy, of the same ethnic group, age and HIV status. Other strengths of the study are the inclusion of the normotensive group to draw comparisons to a matched control cohort and simultaneous characterization of microbiomes in three distinct body sites.

A limitation of this study includes the small sample size used for statistical analysis due to the exclusion of samples with insufficient sequencing data. To control for inter-individual variability, samples needed to be as homogenous as possible. However, by excluding for women who presented with symptomatic infection (vaginal and oral infection), it could possibly have excluded some species that could be implicated in the pathogenesis of pre-eclampsia. The absence of asymptomatic BV in the participants of both cohorts was not confirmed using laboratory methods and should be taken into account with the results reported in this study. A limitation of the study is therefore that the exclusion or diagnosis of BV was not confirmed amongst participants of both cohorts and therefore it is not known whether BV had a contributing role to the observed vaginal microbiome in the pre-eclampsia cohort and weather BV could have increased the risk of these women to develop pre-eclampsia. Another limitation was the lack of the captured medical information of the enlisted participants, such as body mass index (BMI), white blood cell (WBC) counts and birth outcomes.

## Conclusion

The current study provided a diversity analysis of the gut, vaginal and oral microbiomes in a cohort of South African pregnant women with and without pre-eclampsia. Pregnant women with a vaginal microbiome characterized by a significant reduction in *Lactobacillus* spp., and dominance by *L. iners* could have an instable vaginal microbiome and that may increase the risk of developing pre-eclampsia. Diversity and compositional analysis have revealed important data that are otherwise scanty on the gut, oral and vaginal microbiomes in pregnant women (predominantly black African) with pre-eclampsia in South Africa. In future research, larger cohorts with functional and longitudinal analysis of the gut, vaginal and oral microbiomes may reveal additional changes across these microbial communities in pregnant women with pre-eclampsia.

## Data availability statement

The datasets presented in this study can be found in online repositories. The names of the repository/repositories and accession number(s) can be found below: NCBI SRA database, accession number PRJNA798597 (BioProjectID).

## Ethics statement

The studies involving human participants were reviewed and approved by Research Ethics Committee of the Faculty of Health Sciences, University of Pretoria, Pretoria, South Africa (116/2016) and the Human Research Ethics Committee of the University of the Witwatersrand, Johannesburg, South Africa (M160573). The patients/participants provided their written informed consent to participate in this study.

## Author contributions

JG is the project leader and was involved in the concept design, sample collection, laboratory work, data analysis, and writing of the manuscript. MK is the principal investigator and grant holder. MK, MR, and ME were involved in the conceptual design of the study as well as the review of the manuscript. HL was involved in the conceptual design of the study, assisted in sample collection, and contributed toward the clinical aspect of the study. DC was involved by providing support for data analysis. All authors read and approved the final manuscript.

## Funding

Financial support was provided by the NHLS Research trust.

## Conflict of interest

The authors declare that the research was conducted in the absence of any commercial or financial relationships that could be construed as a potential conflict of interest.

## Publisher's note

All claims expressed in this article are solely those of the authors and do not necessarily represent those of their affiliated organizations, or those of the publisher, the editors and the reviewers. Any product that may be evaluated in this article, or claim that may be made by its manufacturer, is not guaranteed or endorsed by the publisher.
