# Supplementary material for: Diversity of the gut, vaginal and oral microbiome among pregnant women in South Africa with and without pre-eclampsia
Source: Front Glob Womens Health. 2022 Sep 16;3:810673. doi: 10.3389/fgwh.2022.810673 (PMC9525020; doi:10.3389/fgwh.2022.810673)
Supplement: Supplementary file 1 [file Table_1.DOCX]

Supplementary Material

1. **Materials and Methods:**

**1.1 Bacterial genomic DNA extraction:**

The following modifications was done in addition to the manufacturer’s instructions when isolating genomic bacterial DNA (gDNA). One millilitre of the seeded PBS was used for gDNA extraction and centrifuged (Labnet Prism^TM^ microcentrifuge, Labnet International) for 5 minutes at 13 000 *x g* to pellet bacterial cells. The pellet was resuspended in a calculated volume of 196 μL of the recommended lysis buffer (Bioline, Taunton, MA), which was prepared for hard-to-lyse bacteria and consisted of TE buffer (20 mM Tris/HCl; 2mM EDTA) and 1% Triton X-100 at a pH of 8. The lysis buffer was supplemented with a cocktail of enzymes for optimal lysis of different bacterial cell wall types. The following enzymes were added: 20.75 μL of lysozyme (10 μg/μL; Sigma-Aldrich, St. Louis, MO), 6.25 μL of mutanolysin (10 U/μL)(Sigma-Aldrich) and 1.67 μL of lysostaphin (3 U/μL in 3M sodium acetate)(Sigma-Aldrich) and incubated at 37°C (AccuBlock ^TM^ Digital Dry Bath, Labnet International) for 60 minutes (Bai et al., 2012; Ravel et al., 2011). Pre-lysis was completed when 25 μL of proteinase K (22 μg/μL; Bioline) was added followed by incubation (56°C for 2 hours). Cell lysis and washing steps were done according to the manufacturer’s instructions and 50 μL of DNA was eluted twice after incubation at room temperature (25°C ± 5°C) for 3 minutes and centrifugation for 1 minute at 11 000 *x g*. Isolated gDNA was quantified and normalized before amplification of the targeted regions.

**1.2 PCR conditions and reagent volumes:**

A single PCR reaction consisted of 12.5 μL of 2x KAPA HiFi DNA HotStart Ready Mix (Kapa Biosystems, Wilmington, MA, USA), 2.5 μL of the gDNA template normalised to 10 ng/μL with 10 mM Tris (pH 8.5) and 5 μL of 1 μM of the forward and reverse primer. The PCR programme comprised an initial denaturation at 95°C for 3 minutes, 25 cycles of denaturation at 95°C for 30 seconds, annealing at 57°C, elongation at 72°C for 30 seconds and one cycle of final extension at 72°C for 5 minutes. Amplification products were analysed using a 2% (m/v) SeaKem (Seakem, Lonza, Switzerland) agarose gel and visualised under ultra-violet light in a BIO-RAD Gel Doc^TM^ EZ Imager (Bio-Rad Laboratories, Hercules, California, USA).

**1.3 Purification of amplified products:**

The volume of the amplified products was adjusted to a final volume of 50 μL with nuclease-free water and mixed with two volumes of binding buffer CB (Bioline, Taunton, MA, USA). The sample was loaded onto the column followed by an incubation step at room temperature (25°C ± 5°C) for 5 minutes. Washing steps were repeated and 30 μL of pre-heated elution buffer (70°C) was added and the column was incubated at room temperature (25°C ± 5°C) for 5 minutes. The ISOLATE II PCR and Gel Column (Bioline, Taunton, MA, USA) was centrifuged at low-speed (1 000 *x g*) followed by another elution step and high-speed centrifugation (11 000 *x g*) for optimal recovery of the DNA template.

**1.4 Creating sequencing libraries**

To construct the sequencing libraries, dual indices and Illumina sequencing adapters were added to the amplified template in an Index PCR by using the Nextera XT Index Kit (Illumina, USA). The Index PCR followed the dual indexing principle and was done according to the Illumina 16S metagenomic library preparation protocol available online. The amplified products were purified using the Agencourt AMPure XP bead-based system (Beckman Coulter, USA). Library quantification was done using a Qubit 3.0 fluorometer (Thermo Scientific, Waltham, MA, USA) to determine the concentration of each sample prior to library normalisation and pooling. The denatured library and the PhiX Control were loaded onto a MiSeq reagent cartridge for sequencing.
